# Transplantation of a free fillet flap from discarded fingers for repair of a finger pulp skin defect: a case report

**DOI:** 10.3389/fsurg.2024.1363827

**Published:** 2024-03-26

**Authors:** Xianting Zhou, Chenxi Zhang, Xuekai Fan, Xiaoming Cai, Xin Wang, Jiadong Pan

**Affiliations:** Department of Plastic Reconstructive Surgery & Hand Microsurgery, Ningbo No. 6 Hospital, Ningbo, China

**Keywords:** finger pulp defect, finger pulp reconstruction, hand trauma, spare-parts, surgical skills

## Abstract

**Background:**

Replantation represents a treatment option for patients with severed finger pulps. However, in some cases, replantation is a challenging task.

**Case presentation:**

We report a successful case of finger pulp reconstruction of the ring finger using free flaps from a nonreplantable index finger in a spare-parts procedure. A 43-year-old worker accidentally injured the index, middle and ring fingers of his left hand on a machine turntable. The severed index and middle fingers and the distal pulp of the ring finger could not be replanted *in situ* due to extensive contusion of blood vessels and soft tissues. After vascular and nerve anastomosis, a free skin flap isolated from the nonreplantable index finger was transplanted to the wound of the distal pulpal defect of the ring finger. The flap survived completely postoperatively. Six months after the operation, only a slight deformity of the ring finger was observed. Moreover, sensation of the digit recovered well.

**Conclusions:**

Spare-part surgery is a surgical approach that effectively saves and utilizes tissue that would otherwise be discarded in cases of severe limb trauma. This idea may be applied to treatment of severe injuries to multiple fingers. Additionally, in the process of tissue transplantation and repair, attention should be given to protecting the tissue in the recipient area to avoid damage to the original undamaged tissue structure, which can adversely affect healing and recovery of the tissue.

## Introduction

For patients with skin defects of the distal pulp of the finger with exposed bone and tendon, a skin flap should be utilized as soon as possible during wound repair (1). The ideal treatment plan must consider good sensation and finger shape (2). Each case presents with a unique mechanism of injury and tissue loss patterns. In general, restoring the effective function and appearance of the hand is a challenge for surgeons.

When tissue from a donor site is needed for reconstruction, it may be in the form of a single tissue graft or a vascularized composite flap. These operations always involve invading the original site of the body, increasing donor site complications (3). Surgeons need to compromise between reestablishing the function of an injured limb and the complications of harvesting tissue from an uninjured donor site.

In addition, due to the unique compartmental structure of the finger pulp, the distal pulp is rich in nerve endings, which cannot be completely replaced by any other tissue (4).

One strategy is to use fillet flaps from an unsalvageable finger provided adequate soft tissue cover for the adjacent fingers. Use of spare-parts fillet flaps in the form of pedicled flaps (5) or free flaps (6) to repair hand skin defects has been reported in many studies.

However, use of free fillet finger flaps in finger pulp skin defect closure is uncommon. Furthermore, how recipient vessels are selected when using the free fillet flap to repair finger wounds has never been reported in detail in previous literature.

We report a case of a 43-year-old handicraftsman who had avulsion severing of the index and middle finger and avulsion of the distal pulp of the ring finger of the left hand.

## Case report

A 43-year-old male patient injured the index, middle and ring fingers of his left hand on a machine turntable. The index and middle fingers were completely severed and severely contused and could not be replanted *in situ*, and the fingers were amputated at the distal 1/3 of the proximal phalanx.

**Figure 1:**
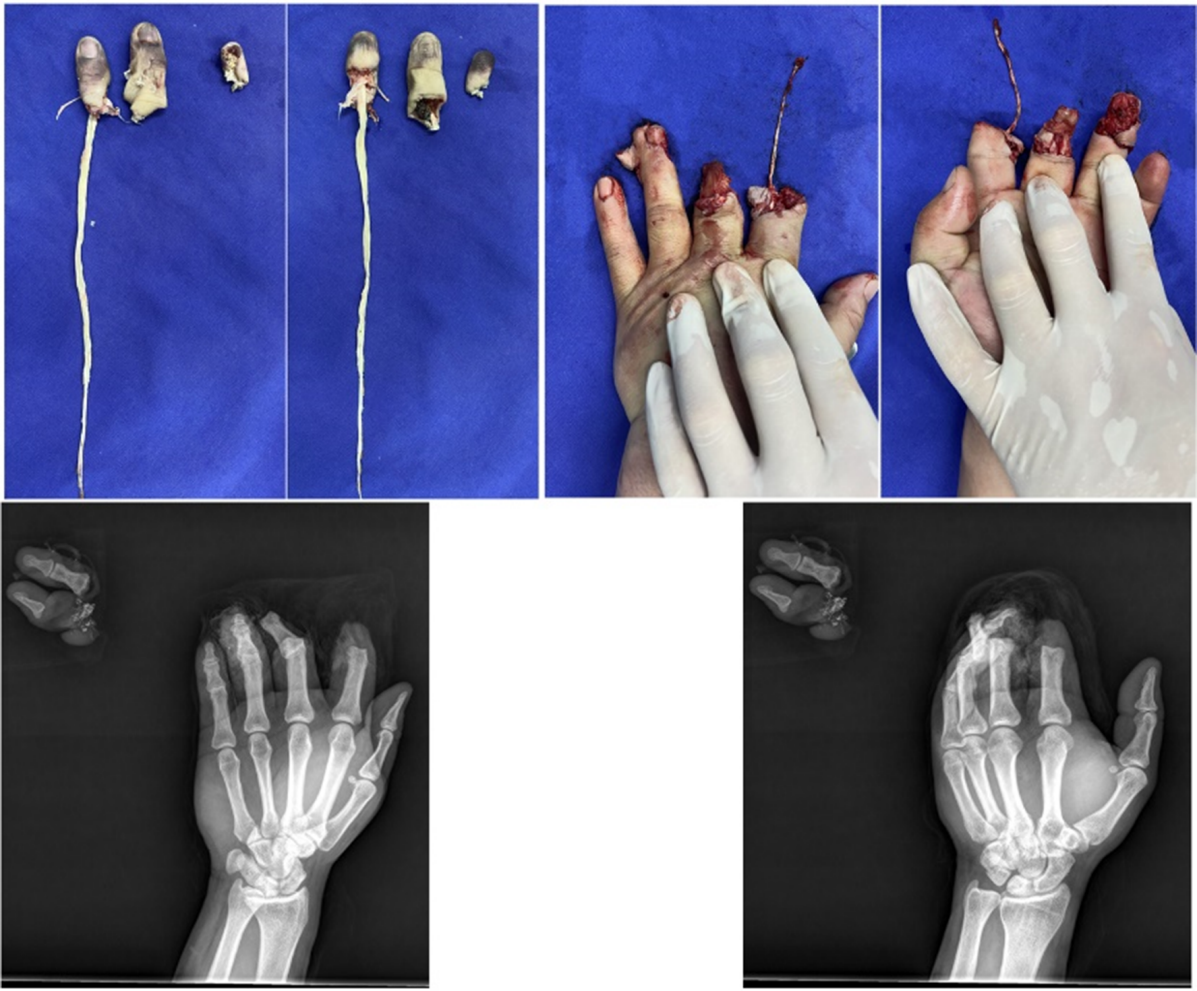
The middle finger was avulsed, the pulp of the ring finger was severed, and ecchymosis was widespread on the severed finger.

The distal pulp of the ring finger was severed with extensive ecchymosis and could not be replanted. The insertion point of the flexor tendon and the distal phalanx of ring finger were exposed, and the defect area was approximately 1.8 cm*2.2 cm (Figure 1).

**Figure 2:**
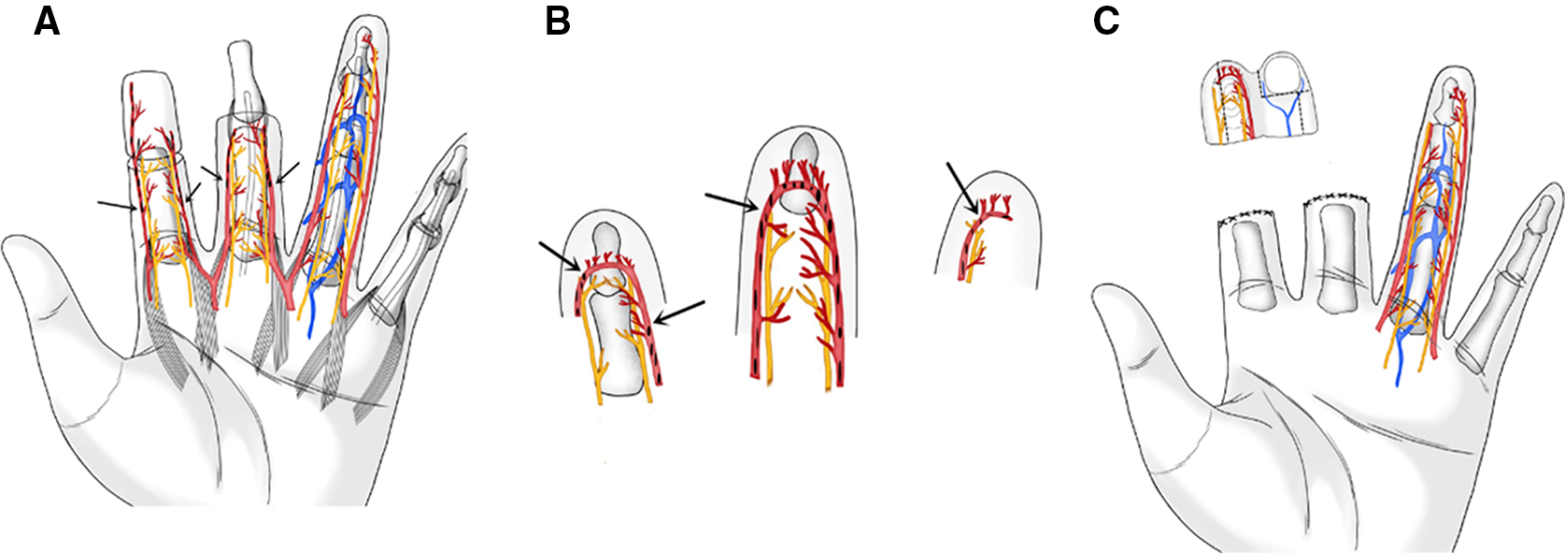
(**A**) Schematic of proximal wound surface. Vascular contusion at Arrow position. (**B**) Schematic diagram of severed finger body. Vascular contusion at Arrowhead position. (**C**) Index finger flap design. The dotted line is the flap clipping line.

Microscopy showed that the ulnar digital artery and nerve were intact and continuous in the wound on the ring finger; the radial digital artery and nerve were severed at the level of the distal interphalangeal joint. A superficial subcutaneous vein was detected at the proximal volar side of the wound (Figures 2A,B).

The distal radial digital artery of the index finger was proximal to the arterial arch with severe contusion and intravascular thrombosis. The ulnar digital artery was complete and continuous beyond the distal interphalangeal joint, a superficial subcutaneous vein was exposed at the dorsal skin edge of the finger, and the bilateral digital nerves were continuous.

The skin was incised along the radial midline of the distal end of the index finger, the volar and dorsal skin was completely excised along the bony surface of the distal phalanx, and the nail bed tissue was excised. The flap area was approximately 4.8 cm*2.5 cm.

**Figure 3:**
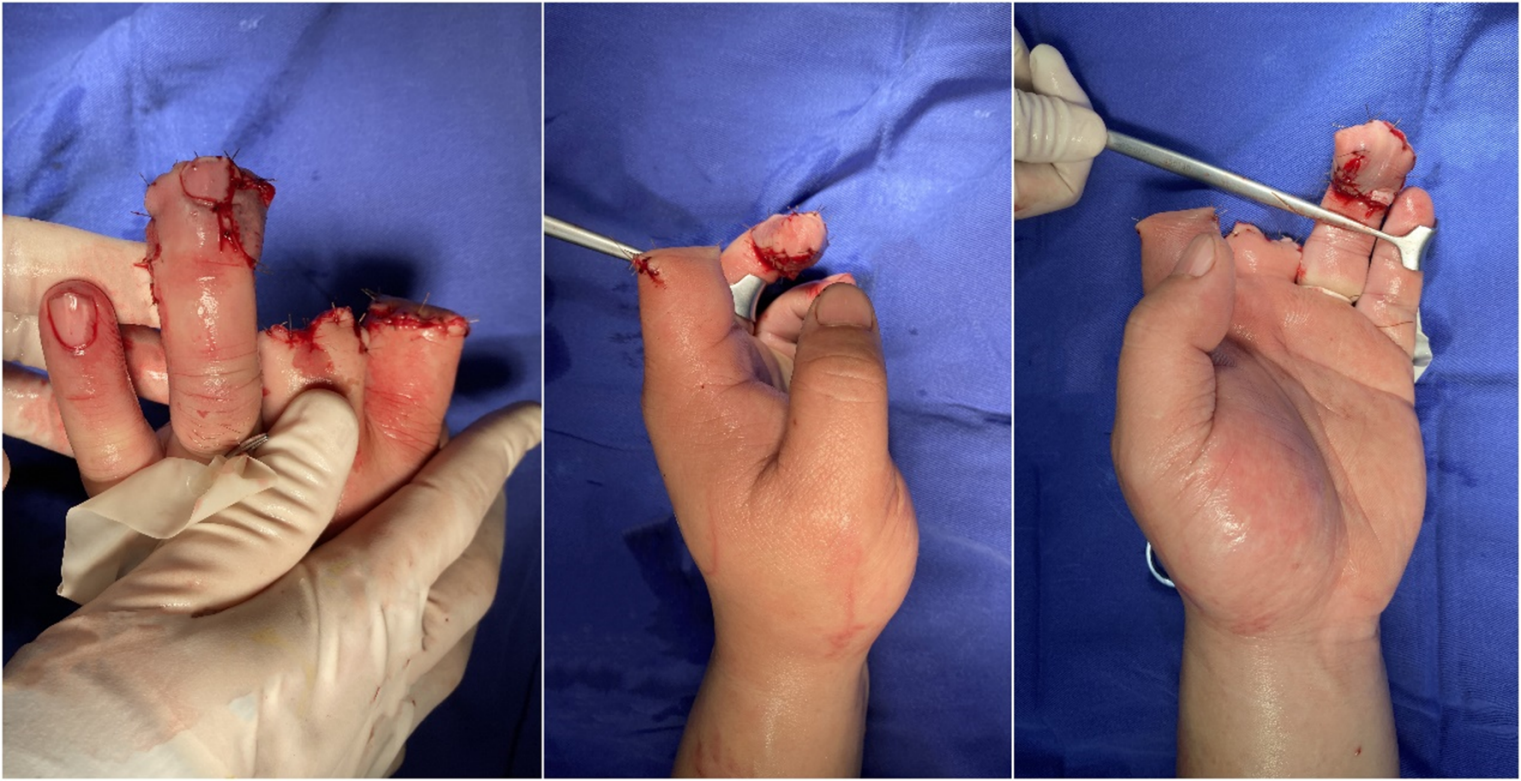
The skin flap was used to repair the finger after operation.

The ulnar digital artery in the free index finger fillet flap was aligned with the radial digital artery of the ring finger. The radial digital nerve in the flap located on the dorsal side of the finger was preserved; as the flap was obviously bloated, it was excised when the flap was trimmed. The trimmed skin flap completely covered the wound, with an area of approximately 2.2 cm*2.5 cm (Figure 2C). The ulnar digital artery and nerve of the index finger were anastomosed with the radial digital artery and nerve of the ring finger, the dorsal vein of the index finger was anastomosed with the palmar vein of the ring finger, and the ring finger defect wound was repaired (Figure 3).

The patient was placed on strict bed rest for 5 days after the operation, during which the flaps were clinically monitored every hour, and antibiotics (cefmetazole, 1 g every 24 h, 3 consecutive days after surgery) were given to prevent infection. Low-molecular-weight heparin was used for anticoagulation, papaverine was injected to prevent vasospasm, and heating of the affected limb with a lamp and oxygen inhalation were performed. The flap survived successfully after surgery.

**Figure 4:**
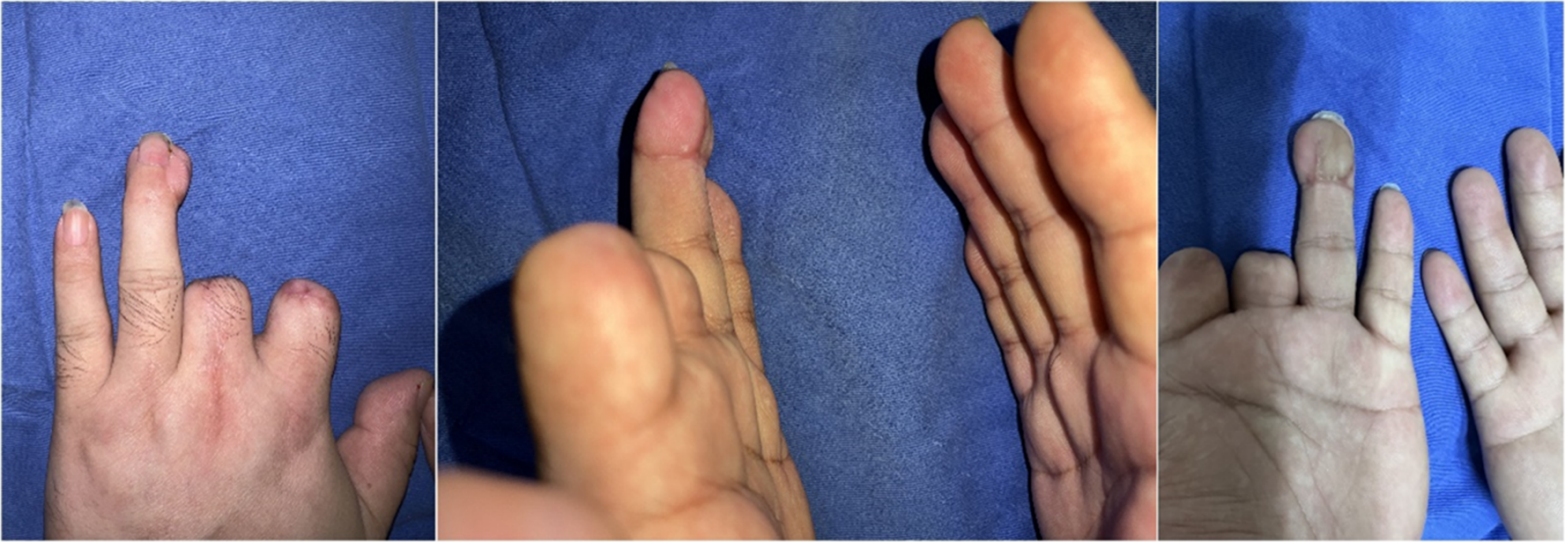
The patients was followed up for 60 days after flap repair.

The patient was hospitalized for 10 days and plaster immobilization was removed at 6 weeks. The patient was able to use the affected limb skillfully at 60 days after the operation and returned to his original life and work (Figure 4). At six months after the operation, the static two-point discrimination sense was 5 mm, the dynamic two-point discrimination sense was 4 mm, and there was good cold resistance and no tenderness. In addition, there was no significant difference in the range of motion between the injured and normal fingers. The patient was satisfied with the appearance and sensation of the repaired finger.

## Discussion

In severe hand trauma, the initial surgical treatment plan is critical for the final outcome (7). For finger amputations, replantation represents the ideal treatment (8). Another treatment option is to remove tissue from other parts of the body for repair and reconstruction when the injured limb tissue is missing. Surgeons often need to balance the function gained after reconstruction of the injured limb against complications in the uninjured donor site.Hentz proposed that when multiple fingers are injured, the available tissue structure of severely injured fingers should be used for repair and reconstruction of other damaged fingers, which improves the recovery function of the remaining fingers (9). This concept is known as “spare parts”; even when the limb is severely injured, the internal tissue structure that can still be used is preserved as much as possible and used for repair and reconstruction of the limb injury at the same time or at a later stage (2). The purpose is to restore limb shape and function as soon as possible and minimize the number of operations and complications at the donor site (10).

Baccarani et al. proposed that a spare-parts fillet flap should be used when local tissue is unsuitable for performing initial closure and when residual limb length is needed (11). Because the ring finger is very important for the grasping function of the hand, we tried to keep the length of the ring finger as long as possible during the operation (2, 12).

Alpert et al. used a free flap from the body of the middle phalanx of the nonreplantation ring finger to repair a skin defect of the middle phalanx of the index finger (13). In our case, the blood supply of the flap was reconstructed, as was the sensation. Sensation recovery is very important for postoperative recovery (14).

Terán et al. used free pulp flaps isolated from nonreplantable severed fingers to repair the defect wounds of other damaged fingers (15). However, injury to the middle finger artery in the ring finger wound and anastomosis between the flap and the ring finger were not described in detail.

In this case, only the ulnar digital artery and nerve were intact in the index finger osteotomy flap. Moreover, the ulnar digital artery and nerve of the ring finger wound were intact and continuous, while the radial digital artery and nerve were severed. Therefore, the ulnar digital artery nerve of the flap was anastomosed to the radial digital artery of the ring finger. The reconstructed skin of the ring finger pulp, including the dorsal and volar skin of the index finger, was inferior to that of the whole finger pulp flap. If the ulnar phalangeal artery of the index finger is anastomosed with the ulnar phalangeal artery of the ring finger, the shape of the affected finger after the operation is better than that of the approach we used.. But it will damage the originally intact ulnar digital artery and increase the occurrence of complications such as cold intolerance after finger surgery (16). In the case of severe trauma to multiple fingers, we not only need to fully evaluate the nonreplantable fingers and use available tissue structure to repair and reconstruct other damaged fingers but also consider protection of the recipient tissue. In this way, primary repair of the defective tissue structure can be achieved, and the complications arising from isolating the needed tissue from other parts of the body can be avoided. This method can also avoid damage to the original undamaged tissue structure in pursuit of repair, which will adversely affect the healing and recovery of the tissue in the recipient area.

## Conclusions

Therefore, in wound repair, we need to fully evaluate the nonreplanted finger according to the injury mechanism and tissue loss of the patients and use the available tissue structure to repair and reconstruct other damaged fingers to achieve one-stage surgical repair of wounds, avoid donor site complications, and pay attention to evaluation and protection of the recipient tissue.

## Data Availability

The raw data supporting the conclusions of this article will be made available by the authors, without undue reservation.
